# Three‐Dimensional Limb Kinematics in Brown‐Throated Three‐Toed Sloths (*Bradypus variegatus*) During Suspensory Quadrupedal Locomotion

**DOI:** 10.1002/jez.2911

**Published:** 2025-03-03

**Authors:** Angela M. Mossor, Andrew J. McKamy, Melody W. Young, Andrew J. Rochté, Judy A. Avey‐Arroyo, John A. Nyakatura, Michael C. Granatosky, Michael T. Butcher, Jesse W. Young

**Affiliations:** ^1^ Kent State University Kent Ohio USA; ^2^ Northeast Ohio Medical University Rootstown Ohio USA; ^3^ Youngstown State University Youngstown Ohio USA; ^4^ Department of Anatomy New York Institute of Technology, College of Osteopathic Medicine Old Westbury New York USA; ^5^ Department of Biology Duke University Durham North Carolina USA; ^6^ Department of Biomedical Engineering Duke University Durham North Carolina USA; ^7^ The Sloth Sanctuary of Costa Rica, Penshurst Limon Costa Rica; ^8^ Dallas World Aquarium Dallas Texas USA; ^9^ Humboldt Universität zu Berlin Berlin Germany; ^10^ Center for Biomedical Innovation, New York Institute of Technology, College of Osteopathic Medicine Old Westbury New York USA; ^11^ Duke Lemur Center Duke University Durham North Carolina USA

**Keywords:** arboreal, biomechanics, joint angles, Xenarthra

## Abstract

Suspensory locomotion differs significantly from upright quadrupedal locomotion in mammals. Nevertheless, we know little concerning joint kinematics of suspensory movement. Here, we report three‐dimensional kinematic data during locomotion in brown‐throated three‐toed sloths (*Bradypus variegatus*). Individuals were recorded with four calibrated high‐speed cameras while performing below‐branch locomotion on a simulated branch. The elbow (range 73°–177°; mean 114°) and knee (range 107°–175°; mean 140°) were extended throughout support phase, with elbow extension increasing with speed. Both the fore‐ and hindlimb displayed abducted proximal limb elements (i.e., arm and thigh) and adducted distal elements (i.e., forearm and leg) during all support phase points. Comparisons of elbow and knee angles between brown‐throated three‐toed sloths and Linnaeus's two‐toed sloths (*Choloepus didactylus*) showed that brown‐throated three‐toed sloths had significantly more extended joint positions during all support phase points. Additionally, across all kinematic measurements, brown‐throated three‐toed sloths showed significant differences between homologous fore‐ and hindlimb segments, with the knee being more extended than the elbow and the arm being more abducted than the thigh. These results are consistent with previously established morphological and behavioral differences between extant sloth genera, with three‐toed sloths showing significantly longer forelimbs than hindlimbs and typically favoring locomotion on angled supports. Our findings show that, despite overall similarities in the use of below‐branch quadrupedal locomotion, the two sloth lineages achieve this locomotor mode with differing kinematic strategies (e.g., degree of joint flexion). These differences may be attributed to the distinct evolutionary pathways through which obligate suspensory locomotion arose in each lineage.

## Summary

- Comparisons of elbow and knee angles between three‐toed and two‐toed sloths showed that three‐toed sloths had more extended joint positions during support phase.
- These differences may be attributed to the evolutionary pathways through which obligate suspensory locomotion arose.

## 1 Introduction

**Figure 1:**
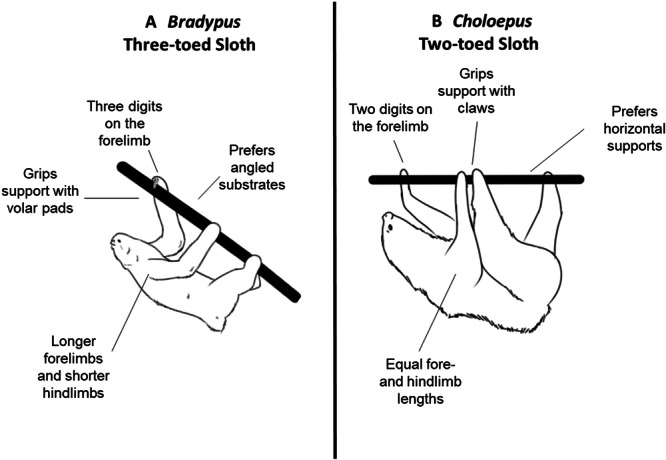
Morphological and behavioral differences between tree sloths. Summaries of previously documented differences in morphology and locomotor behavior between (A) *Bradypus* and (B) *Choloepus*.

Quadrupedal suspensory locomotion has evolved at least 14 times across extant mammals (Fujiwara et al. 2011). However, extant tree sloths (four species of *Bradypus* and two species of *Choloepus*) are the only mammals for which these suspensory habits are near obligatory (Hayssen 2009, 2010; Nyakatura 2012; Nyakatura and Andrada 2013; Slater et al. 2016). Despite general anatomical similarities, the two genera of tree sloths have distinct morphological features and ecological preferences that could influence locomotor mechanics (see Figure 1: Adam 1999; Hayssen 2009, 2010, 2011). Two‐toed sloths (*Choloepus*) have fore‐ and hindlimbs that are of near equal length (Wislocki 1928; Meritt 1985) and elongate, hook‐shaped feet (Mendel 1981a, 1981b) with two unfused digits on the forefeet (Mendel 1985a, 1985b). In comparison, three‐toed sloths (*Bradypus*) have elongated forelimbs with three partially fused digits on the forefeet (Mendel 1985a, 1985b), paired with relatively shorter hindlimbs and hindfeet with a long calcaneus and short metatarsals (Marshall et al. 2021). These interspecific anatomical variations could affect support interactions during suspensory locomotion, and, accordingly, limb kinematics (Meldrum et al. 1998; Godfrey et al. 2016; Granatosky 2022).

Based on previously published data on limb kinematics and kinetics for suspensory locomotion in Linnaeus's two‐toed sloth (*Choloepus didactylus* (*C. didactylus*)) (Nyakatura et al. 2010; Nyakatura and Fischer 2010; Granatosky and Schmitt 2017; Granatosky, Karantanis, et al. 2018; Dickinson et al. 2022.), two‐toed sloths are indicated to employ a diagonal‐sequence diagonal‐couplet gait (Nyakatura et al. 2010), where their forelimbs are the main propulsive elements and the hindlimbs act in net braking (Granatosky 2018a; Granatosky, Fitzsimons, et al. 2018; Granatosky and Schmitt 2017, 2019), with equal body weight distribution between limb pairs (Granatosky, Karantanis, et al. 2018). Conversely, brown‐throated three‐toed sloths (*Bradypus variegatus* (*B. variegatus*)) utilize a lateral‐sequence diagonal‐couplet gait during suspensory walking (Mendel 1985a; Gorvet et al. 2020), and recent studies have shown that, while three‐toed sloths also utilize their hindlimbs as a braking element, they exhibit hindlimb‐biased body weight support (McKamy et al. 2023; Young et al. 2023). From these measured variations in gait patterns and limb kinetics, overall limb kinematics are expected to also differ between extant sloth genera.

Despite the numerous observed differences between below‐branch and upright locomotion (Gray 1944; Usherwood and Self Davies 2017; Granatosky 2018b; Granatosky, Fitzsimons, et al. 2018), two‐toed sloths display limb kinematics similar to those reported for small‐ to medium‐sized noncursorial mammals with respect to limb element angles, joint excursions, and the contribution of individual limb segments to forward progression of the body (Nyakatura et al. 2010). However, there are comparatively fewer studies that have examined locomotor mechanics in three‐toed sloths (but see McKamy et al. 2023; Young et al. 2023; Gorvet et al. 2020; Mendel 1985a, 1985b), and none that have focused on their three‐dimensional (3D) limb kinematics.

The aims of this study are to: (1) evaluate and report detailed 3D limb kinematics in brown‐throated three‐toed sloths (*Bradypus variegatus*) to better understand locomotor kinematics in an obligate suspensory mammal; (2) determine how suspensory limb kinematics in brown‐throated three‐toed sloths differ from upright pronograde (above branch) locomotion; (3) to assess the degree to which limb kinematics are convergent or divergent between two‐ and three‐toed sloths.

## 2 Materials and Methods

### 2.1 Animals and Experimental Setup

Data from Linnaeus's two‐toed sloth (mass: 8.13 ± 2.17 kg) were obtained from Nyakatura et al. (2010). We only report elbow and knee angles here because they are the only values for which 3D data were available in this pre‐existing data set.

Four adult brown‐throated three‐toed sloths (mass: 3.88 ± 0.3 kg) were used in this study. Sloths were selected and handled mainly by staff at the Sloth Sanctuary of Costa Rica (Penshurst, Limon, Costa Rica). All animals were healthy with no visible signs of musculoskeletal or gait abnormalities, and no preference was given to male or female individuals (three male and one female). All experimental procedures complied with the protocols approved by the Costa Rica Ministerio Del Ambiente y Energía, Sistema Nacional de Áreas de Conservación, a través del Programa de Investigación del Área de Conservación La Amistad Caribe, (R‐SINAC‐PNI‐ACLAC‐012‐2021 to M. T. Butcher).

**Figure 2:**
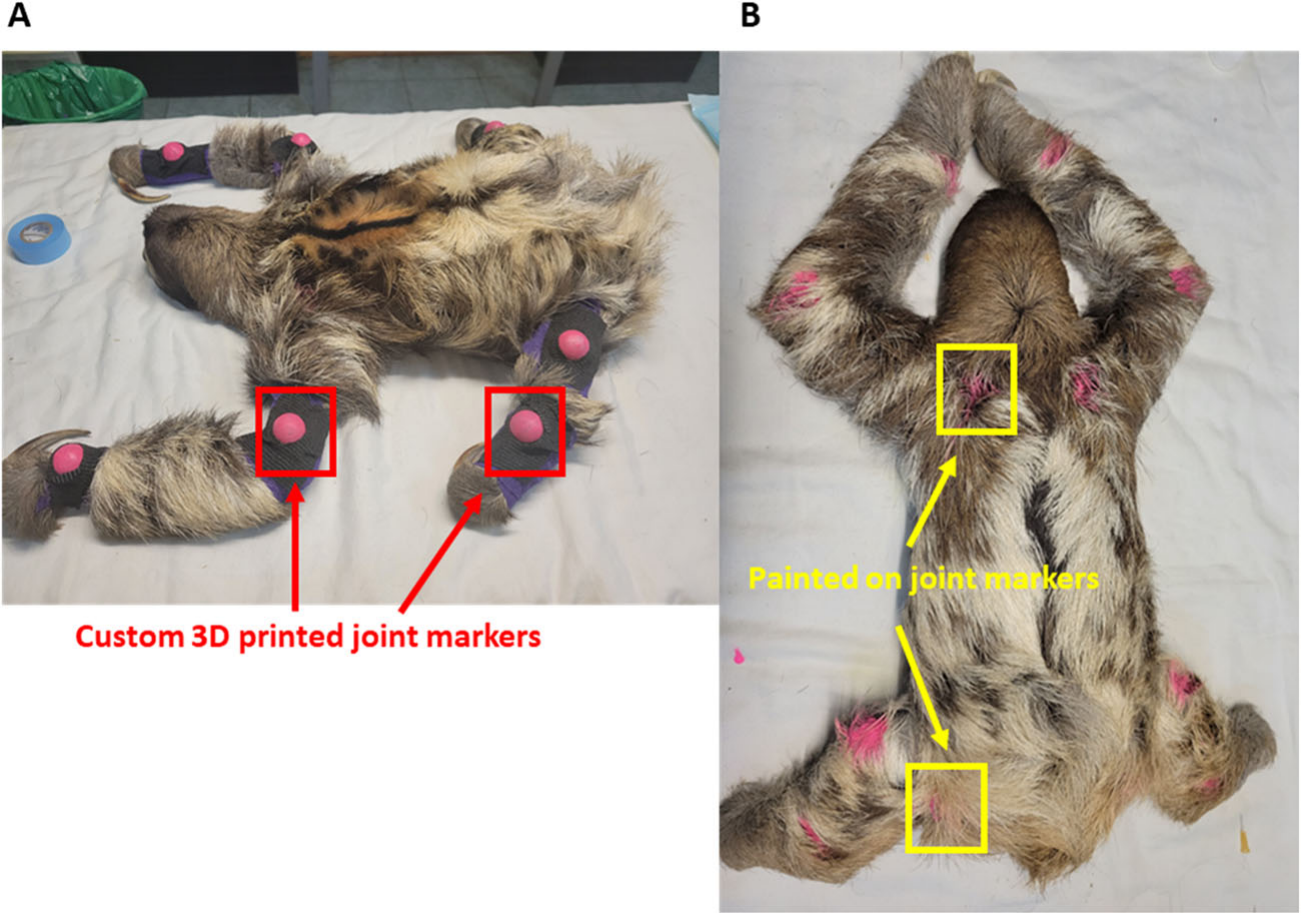
Exemplar joint marker placements. Examples of (A) 3D printed joint markers placed on the distal limb elements (wrist, elbow, ankle, and knee), and (B) fluorescent painted markers for the proximal limb elements (shoulder and hip).

Prior to data collection, animals were sedated with Dexdomitor (0.5 mL kg^−1^, injected into the left *M. gluteus medius*) for joint marker placement. 3D printed joint markers (10 mm in diameter) that were marked with fluorescent paint were attached to the lateral surfaces of the elbow, wrist, knee, and ankle with vet wrap, in a way that did not restrict joint movement, for kinematic tracking and analyses (Figure 2A). Shoulder and hip landmarks were brushed on with fluorescent, animal‐safe paint to aid tracking (Figure 2B). Additionally, measurements were taken of limb segments between joints to establish marker position more precisely during later analyses (see description below and Table 1). Following marker placement, anesthesia was reversed with Antisedan (0.5 mL kg^−1^ into the left *M. gluteus medius*) and the animal was given time to recover.

**Table 1:** Limb segment measurements (in cm).

Individual	Arm		Forearm		Thigh		Leg	
	Right	Left	Right	Left	Right	Left	Right	Left
BV1	17	17	17.5	17.5	11.5	11.5	11.5	11.5
BV2	16	16	16.5	17	9.75	10	11	10
BV3	18.5	18.5	17.5	17.5	12.5	11.5	12.5	13.5
BV4	18.5	18	18.5	17.5	11	11	11.5	12.5

After recovery, individuals were encouraged to traverse a custom‐built runway beam made of caña brava (~2.9 m long*; Gyneriums agittatum*, Figure 2B) for suspensory locomotion trials, unconstrained and at their preferred speed. Locomotor activity was recorded using four GoPro cameras (HERO10, GoPro, San Mateo, CA, USA) positioned on each side of the runway beam (Figure 2A) at sagittal and diagonal views at a frame rate of 60 Hz. Over 9 sets of sampling days, we collected a total of 84 (44 forelimbs and 40 hindlimbs) strides suitable for kinematic analyses.

### 2.2 Kinematic Calibration and Analyses

Two‐dimensional pixel positions from each camera pair (i.e., left and right relative to the custom‐built runway beam) were calibrated into a 3D coordinate system (in meters) using the protocol of Theriault et al. (2014). Because our analyses focused on individual forelimb and hindlimb strides, we did not merge the left and right sets of cameras into a single system but rather analyzed left and right limbs separately in our analyses, using separately calibrated coordinate systems. In both final coordinate systems, the *x*‐axis was set to coincide with the long axis of the runway beam support (i.e., the fore‐aft direction), the *y*‐axis defined the mediolateral direction, and the *z*‐axis defined the vertical direction. The origin of the system was set to the midpoint of the central axis of the runway beam. For each individual stride, axes were then standardized to the predominant direction of animal movement such that positive *x*‐axis displacement was defined as forward (i.e., cranial) movement, positive *y*‐axis displacement was defined as leftward movement (i.e., relative to the central axis of the beam), and positive *z*‐axis displacement was defined as an upward movement (i.e., opposite of the gravity vector), following the right‐hand rule.

**Figure 3:**
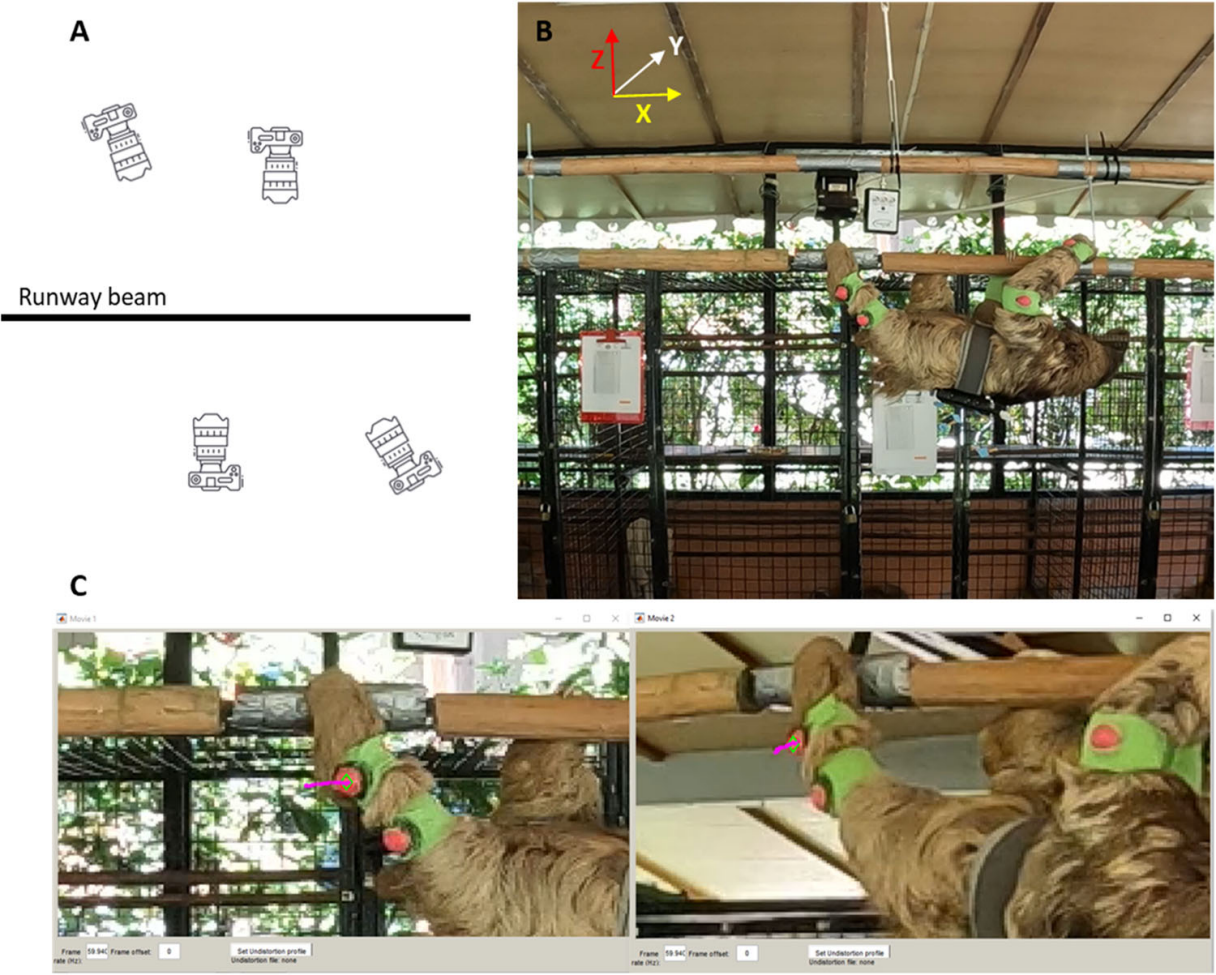
Locomotor runway beam setup and joint marker tracking. (A) Schematic of camera placements relative to the runway beam setup. (B) Visual representation of an individual suspensory walking below the runway beam setup during a trial, shown at the moment of forelimb mid‐stride phase. (C) Example of 3D joint marker tracking of the ankle in two cameras.

Individual joint markers for the fore‐ and hindlimb were digitized and reconstructed in 3D space DLTdv8a (Hedricks 2008) in MATLAB (Mathworks Inc., Natick, MA, USA) (Figure 3C). A total of 14 points were identified and tracked across the individual and runway beam (Table 2). Reconstructed 3D points were then exported as .csv files to be analyzed in a custom MATLAB program (available for download at: 10.6084/m9.figshare.26069551). Raw coordinate data were fit to a quintic smoothing spline function (MATLAB function “spaps”; tolerance of 0.5 cm^2^), allowing us to mitigate digitizing error and interpolate feature positions for frames where the marker was not visible across gaps of ≤ 10% of limb support phase duration. Given that we observed evidence of skin movement in the proximal joint markers (i.e., shoulder, elbow, hip, and knee), we adjusted their apparent position using the following procedure. Beginning with the distal‐most joint (i.e., wrist or ankle, where skin movement was minimal) we used apparent marker position to define a unit vector representing the orientation of the mid‐joint marker (i.e., elbow or knee) relative to the distal marker. We used the inter‐marker distances measured while the animals were anesthetized (see above) to translate marker position along this vector, creating a corrected mid‐joint marker position. We followed the same procedure to fix the position of the proximal‐most marker (i.e., shoulder or hip) relative to the corrected position of the mid‐joint marker. Overall forelimb and hindlimb protraction angles were calculated as the two‐dimensional plane angle between the limb vector (proximal‐most point to distal‐most point) and the global *x*‐axis. Abduction/adduction angles were calculated as the two‐dimensional plane angle between the relevant segment vector the global *y*‐axis. Finally, mid‐joint angles were calculated as the two‐dimensional plane angles between arm and forearm or the thigh and leg limb regions.

**Table 2:** Marker numbers and placements used for video tracking.

Marker number	Marker placement	Point type
1	Shoulder	Dynamic
2	Elbow	Dynamic
3	Wrist	Dynamic
4	Hip	Dynamic
5	Knee	Dynamic
6	Ankle	Dynamic
7	Beginning of substrate	Static
8	End of substrate	Static
9	Head	Static
10	Tail base	Static
11	Forelimb touch down	Time point
12	Forelimb liftoff	Time point
13	Hindlimb touch down	Time point
14	Hindlimb liftoff	Time point

*Note:* Point types are identified as dynamic (tracked in multiple frames throughout support phase), static (tracked in a single frame, nonmoving point), or time points (dummy spatial points used for identifying event timing during subsequent analyses).

Limb support phase events during a stride cycle were defined based on a combination of video coding and marker position analysis. Specifically, forelimb/hindlimb touchdown (TD) was coded as the first frame in the video when the limb of interest contacted the beam following a preceding swing phase. Similarly, liftoff (LO) was defined as the first frame in which the limb did not contact the beam following a preceding support phase. Finally, mid‐stride (MS) was defined as the frame in which the cranio‐caudal (*x*‐axis) position of the proximal‐most joint marker (shoulder or hip) was closest to that of the distal‐most joint marker (wrist or ankle). For each given limb support phase event, values for elbow angle, arm abduction, forearm abduction, forelimb protraction, knee angle, thigh abduction, leg abduction, and hindlimb protraction were calculated. Fore‐ and hindlimb angular excursions during limb support phase were calculated as the difference of limb protraction angles at TD and LO. Overall stride speed was calculated as the mean of the first derivative of the smoothed position data for the proximal‐most joint marker (shoulder or hip) with respect to time. All processed data were then saved and exported as .csv files for statistical analyses.

### 2.3 Statistical Analyses

Final data were qualitatively checked for normality using quantile–quantile plots, following the recommendations of Sokal and Rohlf (2011). Distributions were Box–Cox transformed (Box and Cox 1964) when necessary to improve normality. Mixed‐effects analyses of covariance were used to compare summary kinematic measurements among limbs, joints, and step events, while controlling for the effects of speed, individual animal, and experiment trial number. Tukey post hoc tests determined significant differences in angular position between limb events and between homologous fore‐ and hindlimb segments/joints, controlling for the effects of speed. In cases where a factor showed a significant interaction with speed (i.e., nonhomogenous slopes across factor groups, such as support phase events), we tested for differences among factor levels at the minimum, mean, and maximum values of the overlapping range of speeds common to all factor levels. Additionally, comparable joint angle data for the fore‐ and hindlimb (elbow and knee joints, respectively) from Linnaeus's two‐toed sloth (*C. didactylus*, Nyakatura et al. 2010) was reanalyzed for interspecies comparisons. All statistical analyses were conducted in R (R Core Team 2020) using the add‐on libraries dplyr (Wickham et al. 2021), tidyr (Wickham et al. 2023), lme4 (Bates et al. 2015), lmerTest (Kuznetsova et al. 2017), ggplot2 (Wickham 2016), and emmeans. All custom‐written R scripts used for analysis, along with requisite data files, are freely available for download at (10.6084/m9.figshare.26069554).

### 2.4 Study Limitations

Small sample size is due to animal availability. Having a larger sample size would improve statistical power for resolving variation among species. Whereas five individuals were initially filmed for this study, only four could be used due to the inability to track the other videos (due to low light environments and variable animal cooperation). Approximations of joint markers may have some degree of error/inaccuracy due to the inability to shave limbs for more accurate marker placement. The movement of skin markers relative to underlying bone/joints is also a potential source of error in kinematic analyses (Reinschmidt et al. 1997; Günther et al. 2003; Bauman and Chang 2010; Torres et al. 2011; Andrada et al. 2017). Additionally, due to the difficulty in placement and accuracy of joint markers, estimates of limb long‐axis rotation could not be achieved. While a horizontal runway beam was used in this study based on published methods (Nyakatura et al. 2010; Granatosky and Schmitt 2017; Granatosky, Karantanis, et al. 2018), an angled runway beam would mimic more realistic natural environments for *B. variegatus* (Hayssen 2009, 2010). Lastly, the use of sedatives and reversal drugs may have created possible effects on animal performance during locomotor trials.

## 3 Results

### 3.1 Limb Support Phase Kinematics

**Figure 4:**
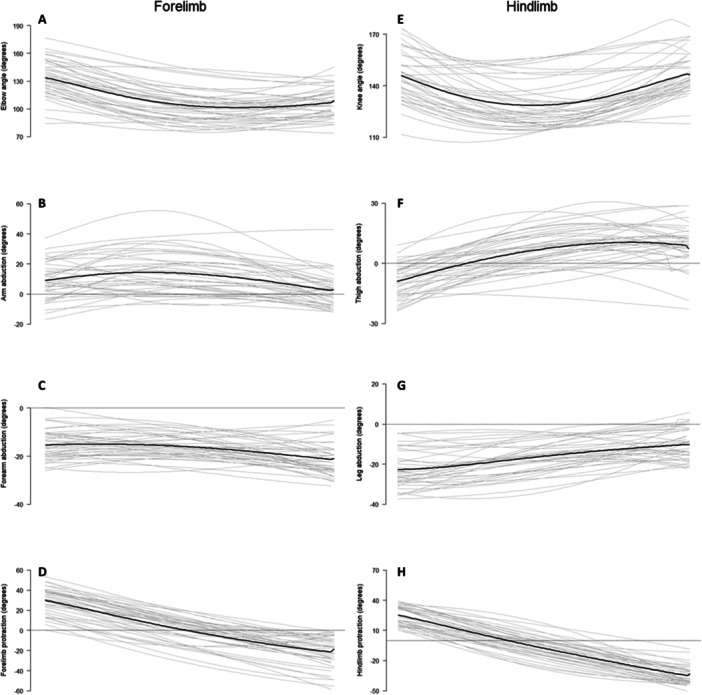
Kinematic profiles for *Bradypus variegatus* during support phase. Light gray lines represent individual kinematic trials, and the bold black line represents the mean kinematic profile across all trials. (A–D) Kinematic profiles for elbow angle, arm abduction, forearm abduction, and forelimb protraction (in degrees). (E–H) Kinematic profiles for knee angle, thigh abduction, leg abduction, and hindlimb protraction (in degrees).

Summaries of forelimb kinematic profiles are presented in Figure 4A–D. At forelimb TD, the elbow is extended (mean: 136.9°), with the arm slightly abducted (mean: 4.2°) and the forearm adducted (mean: −16.2°). Throughout forward progression the elbow flexes (mean MS angle: 102.1°), where the abduction/adduction of the arm (mean MS angle: 5.0°) and forearm (mean MS angle: −18.0°) remain relatively unchanged. At forelimb LO, the elbow is significantly less extended (mean: 105.1°) than during the beginning of support phase (i.e., TD). The arm is still only slightly abducted (mean: 3.41°) and the forearm is even more adducted (mean: −20.7°) relative to limb TD.

Summaries of hindlimb kinematic profiles are presented in Figure 4E–H. At hindlimb TD, the knee is extended (mean: 148.0°), with the thigh slightly abducted (mean: 1.26°) and the leg adducted (mean: −20.3°). During forward progression, the knee becomes less extended (mean MS angle: 125.6°), whereas the abduction/adduction of the thigh (mean MS angle: 2.7°) and leg (mean MS angle: −18.2°) remain relatively unchanged. At hindlimb LO, the knee returns to a similar extended position as that at TD (mean: 147.7°), whereas the thigh is more abducted (mean: 3.9°) and the leg is less adducted (mean: −11.2°) relative to limb TD.

### 3.2 Elbow and Knee Angles

**Figure 5:**
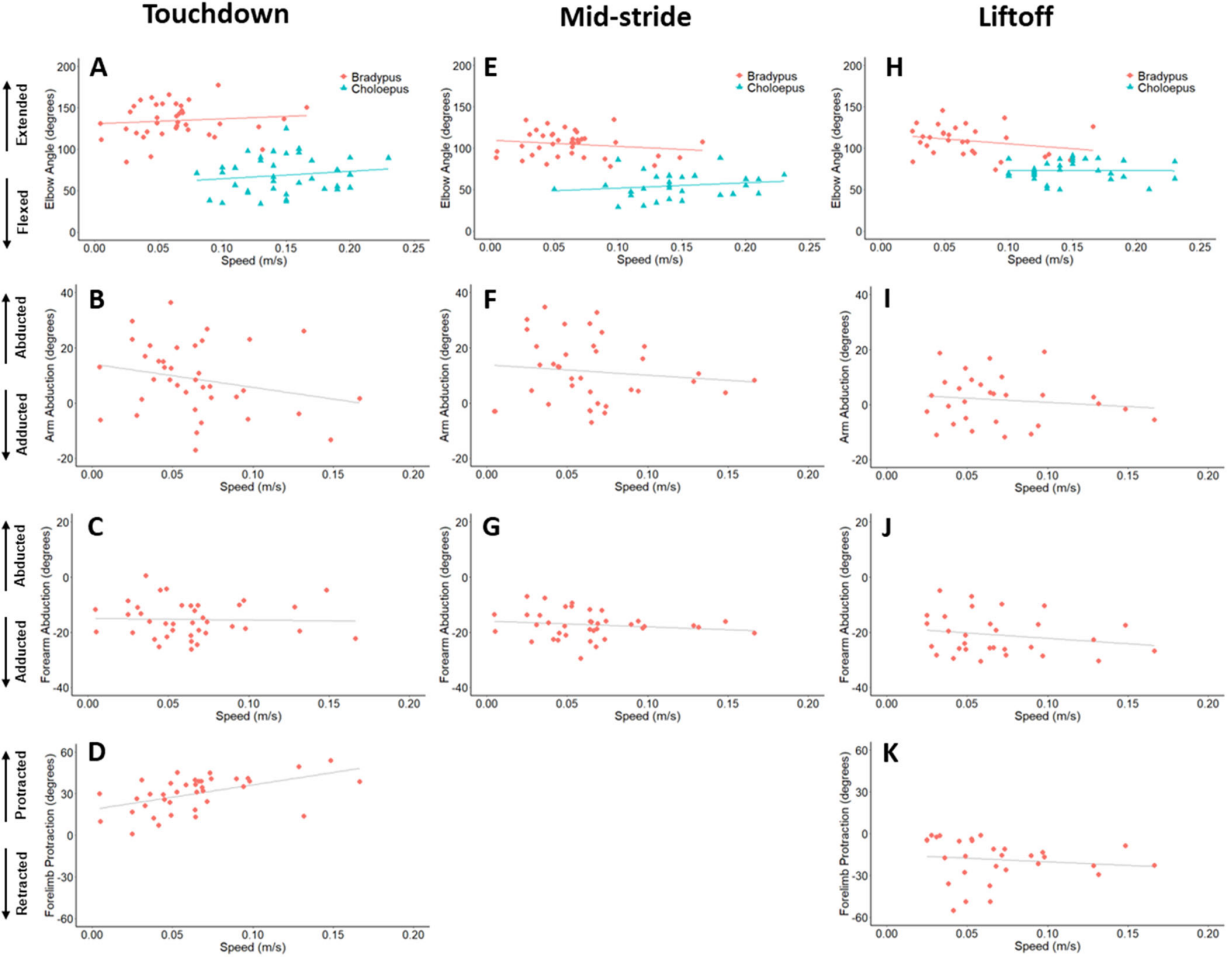
Kinematics (in degrees) of the forelimb during touchdown (TD), mid‐stride (MS), and liftoff (LO) versus speed (in m/s). Light gray statistical trendlines indicate where speed‐related trials are nonsignificant. (A, E, H) Elbow angles for both *B. variegatus* and *C. didactylus*. (B, F, I) Arm abduction values for *B. variegatus*. (C, G, J) Forearm abduction values for *B. variegatus*. (D and K) Forelimb protraction values for *B. variegatus*.

**Figure 6:**
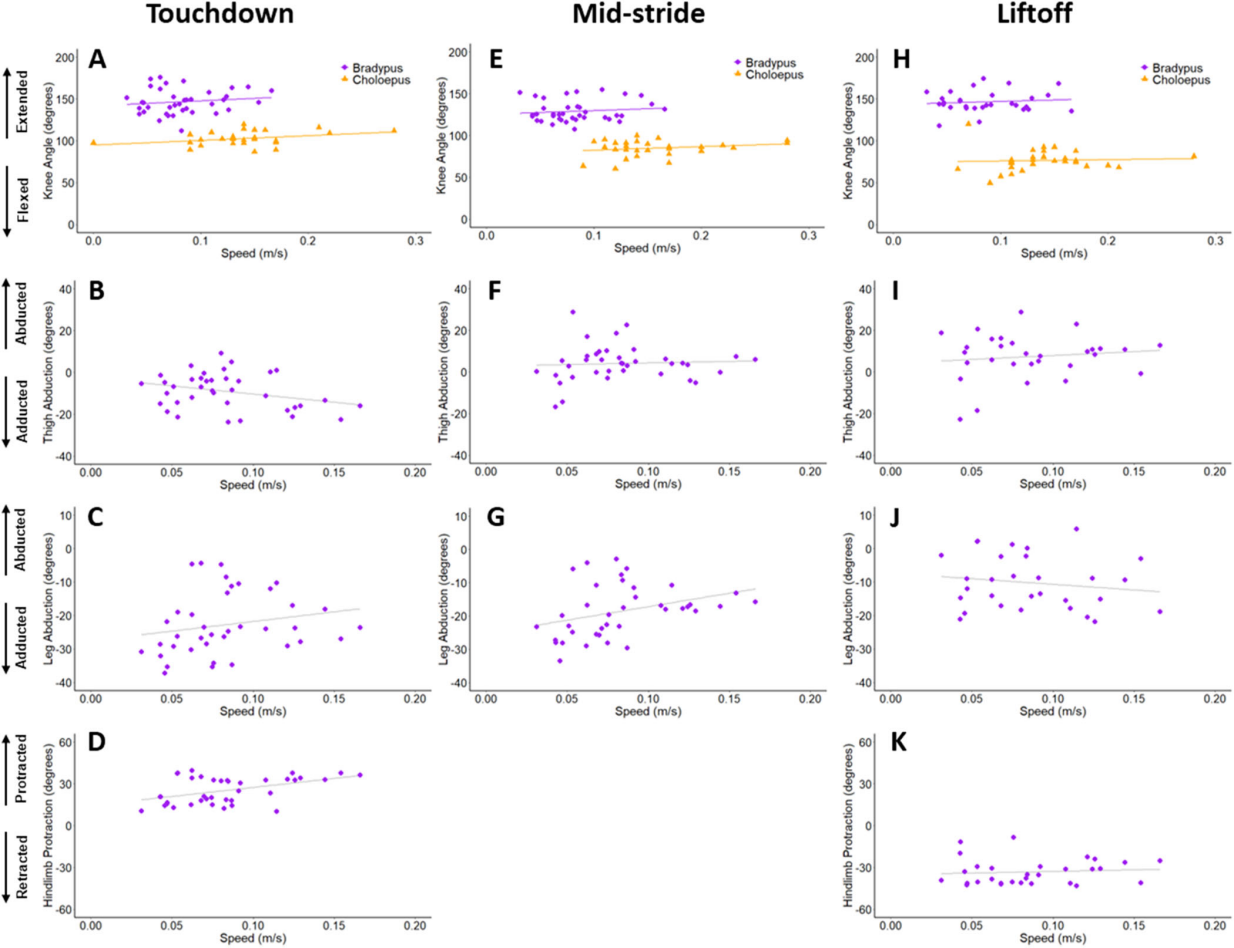
Kinematics (in degrees) of the hindlimb during touchdown (TD), mid‐stride (MS), and liftoff (LO) versus speed (in m/s). Light gray statistical trendlines indicate where speed‐related trials are nonsignificant. (A, E, H) Knee angles for both *B. variegatus* and *C. didactylus*. (B, F, I) Thigh abduction values for *B. variegatus*. (C, G, J) Leg abduction values for *B. variegatus*. (D and K) Hindlimb protraction values for *B. variegatus*.

Variation in elbow and knee angles is displayed in Figures 5A,E,H and 6A,E,H, respectively. Data are shown for both brown‐throated three‐toed sloths and Linnaeus's two‐toed sloth (where available). The outcomes of all statistical tests are summarized in Tables S1 and S2. Brown‐throated three‐toed sloths did not show a significant interaction between speed and limb support phase events for elbow angle (*p* = 0.188). There was a significant main effect for limb support phase event (*p* < 0.001), where the elbow was most extended at TD (*p* < 0.001), relative to both MS and LO. Similarly, there was no significant interaction between speed and limb support phase events for knee angle (*p* = 0.964), however, independent of speed, the knee was the most flexed at MS (*p* < 0.001). There was a nearly significant trend for both elbow and knee extension to increase with speed (*p* = 0.056). Finally, joint angles between the elbow and knee were significantly different (*p* < 0.001) throughout limb support phase, with the knee consistently more extended than the elbow (Table 3).

**Table 3:** Estimated marginal mean values of elbow and knee angles (in degrees) for support.

Species	Support phase event	Mean elbow angle (degrees)	Elbow: lower confidence limit	Elbow: upper confidence limit	Mean knee angle (degrees)	Knee: lower confidence limit	Knee: upper confidence limit
*Bradypus variegatus*	Touchdown	136.9	127.0	146.9	148.0	143.3	152.0
	Mid‐stride	102.1	95.1	109.0	129.8	125.6	134.0
	Liftoff	105.1	98.3	111.8	147.7	143.0	152.0
*Choloepus didactylus*	Touchdown	64.2	52.2	76.2	100	93.5	107
	Mid‐stride	51.6	43.4	59.9	82.4	74.6	90.2
	Liftoff	73.1	63.8	82.5	75.8	68.5	83

*Note:* Phase points in both *Bradypus variegatus* and *Choloepus didactylus*. Confidence limits are based on 95% confidence intervals.

Interspecies comparisons were conducted for both elbow and knee angles between brown‐throated three‐toed sloths and Linnaeus's two‐toed sloth. Overall, both knee and elbow kinematics showed significant effects for both speed (*p* < 0.001) and species (*p* < 0.001). Across the limb support phase, the elbows (*p* < 0.001) and knees (*p* < 0.001) of *B. variegatus* were significantly more extended in comparison to *C. didactylus*. This interspecific difference was also true at each limb support phase event—TD, MS, and LO—for both the elbow and knee, with brown‐throated three‐toed sloths consistently showing more extended limbs overall (all *p* < 0.001*;* see Table 3). Heuristically defining elbow/knee angles over 90° as an extended posture, and those below 90° as a flexed posture, brown‐throated three‐toed sloths consistently used extended postures, compared to the flexed joint postures of Linnaeus's two‐toed sloth.

### 3.3 Transverse Plane Limb Kinematics

Variation in proximal limb segment abduction for both the forelimb (arm) and hindlimb (thigh) is displayed in Figures 5B,F,I and 6B,F,I, respectively, as well as in Table 4. The outcomes of all statistical tests are summarized in Table S3. Both arm and thigh abduction angles were not influenced by speed (*p* ≥ 0.660). However, the main effect for limb support phase event (*p* = 0.003) was significant, where the arm was less abducted at MS (*p* = 0.002) relative to LO. The main effect for limb support phase event for the thigh (*p* = 0.002) was significant, where the thigh was less abducted at TD (*p* = 0.003) than at LO. Differences between arm and thigh abduction varied among events, where the arm was more abducted than the thigh during TD (*p* < 0.001) and MS (*p* < 0.001).

**Table 4:** Estimated marginal mean values (in degrees) of *Bradypus variegatus* for arm abduction, forearm abduction, forelimb protraction, thigh abduction, leg abduction, and hindlimb protraction across all support phase events, along with 95% confidence limits.

Measurement	Support phase event	Mean value (degrees)	Lower confidence limit	Upper confidence limit
Arm abduction	Touchdown	4.17	2.46	5.89
	Mid‐stride	5.02	3.36	6.69
	Liftoff	3.41	1.88	4.95
Forearm abduction	Touchdown	−16.2	−21.3	−11.1
	Mid‐stride	−18.0	−23.1	−12.9
	Liftoff	−20.7	−25.4	−16.0
Forelimb protraction	Touchdown	30.3	22.69	37.91
	Mid‐stride	1.36	−6.24	8.97
	Liftoff	−20.41	−28.02	−12.8
Thigh abduction	Touchdown	1.26	−0.16	2.68
	Mid‐stride	2.67	1.78	3.56
	Liftoff	3.87	2.83	4.90
Leg abduction	Touchdown	−20.3	−41.3	0.69
	Mid‐stride	−18.2	−39.2	2.77
	Liftoff	−11.2	−28.7	6.28
Hindlimb protraction	Touchdown	20.76	−3.82	45.3
	Mid‐stride	−0.54	−25.13	24.0
	Liftoff	−37.91	−61.16	−14.6

Variation in distal limb segment abduction for both the forelimb (forearm) and hindlimb (leg) is displayed in Figures 5C,G,J and 6C,G,J, respectively. The outcomes of all statistical tests are summarized in Table S4. There was no significant relationship between speed and abduction (*p* = 0.703) in the forearm. However, the forearm was less adducted in during limb TD (*p* = 0.009) comparison to LO. There was not a significant interaction between speed and abduction (*p* = 0.660) in the leg. However, event was significant (*p* < 0.001) where the leg was the least abducted at LO. Distal limb segment abduction was significantly different when comparing the fore‐ and hindlimb, where the forearm was more adducted than the legat LO (*p* < 0.001).

### 3.4 Sagittal Limb Kinematics

Variation in limb protraction for both the fore‐ and hindlimb is displayed in Figures 5D,K and 6D,K, respectively, as well as in Table 4. The outcomes of all statistical tests are summarized in Table S5. There was no significant interaction between speed and limb support phase event for forelimb protraction (*p* = 0.159). Event (*p* < 0.001) was significant, where the forelimb was most protracted at TD (*p* < 0.001). There was no significant interaction between speed and limb protraction event in the hindlimb (*p* = 0.245). There was a significant difference in hindlimb protraction based on event (*p* < 0.001), with the limb being most protracted at TD (compared to MS: *p* < 0.001) and retracted at LO (compared to MS: *p* < 0.001). Limb protraction also significantly differed between the fore‐ and hindlimb event interactions where the forelimb was more protracted than the hindlimb at TD (*p* < 0.001), whereas the hindlimb was more retracted than the forelimb at LO (*p* < 0.001).

**Figure 7:**
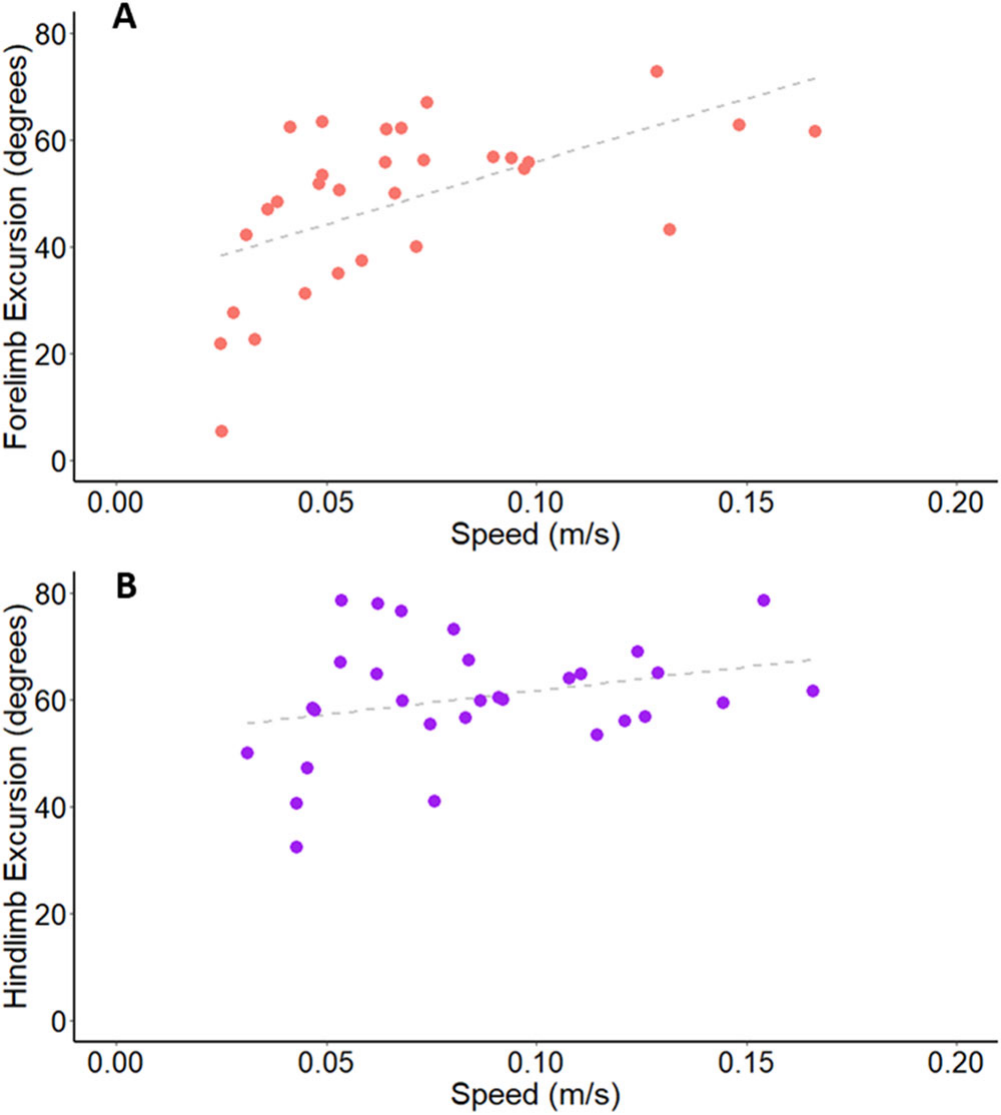
Limb excursions (in degrees) for the forelimb and hindlimb versus speed (in m/s). (A) Forelimb and (B) hindlimb excursions for *B. variegatus* with statistical trendlines.

The relationship between speed and limb excursion is shown in Figure 7. The outcomes of all statistical tests are summarized in Table S6. There was no significant interaction between speed and limb excursion (*p* = 0.977), indicating a similar relationship between speed and excursion across limb girdles. Both fore‐ and hindlimb excursion significantly increased with speed (both *p* values ≤ 0.010), but there was no significant main effect of limb girdle, indicating similar magnitudes of excursion in fore‐ and hindlimbs (*p* = 0.493).

## 4 Discussion

During quadrupedal suspensory locomotion, limbs are positioned such that flexor, rather than extensor muscle activity is largely required to maintain joint position and limb postures (Demes and Carlson 2009; Fujiwara et al. 2011; Nyakatura 2012). Morphology and patterns of muscle activation have evolved to meet these demands by increasing the leverage and masses of flexor versus extensor musculature (Jenkins and Weijs 1979; Tuttle et al. 1983; Jouffroy and Stern 1990; Gregersen et al. 1998; Wickler et al. 2005; Fujiwara et al. 2011; Olson et al. 2018; Gorvet et al. 2020) in suspensory mammals. Brown‐throated three‐toed sloths spend nearly one‐half of their active time in suspension (~ 44% of locomotor repertoire: Urbani and Bosque 2007; Hayssen 2010) and therefore have better developed flexor musculature with larger physiological cross‐sectional area in both the forelimb (Olson et al. 2018) and hindlimb, in comparison to the extensor muscles (Mendel 1985b; Nyakatura and Fischer 2011). Additionally, flexor muscles are more active during suspensory locomotion (Gorvet et al. 2020). Though increased flexor musculature activity and strength is necessary for limb stability when counteracting gravity, this study shows that brown‐throated three‐toed sloths nonetheless exhibit, on average, relatively extended fore‐ and hindlimbs during individual limb support phases during suspensory locomotion, specifically at the elbow and knee joints (i.e., elbow and knee angles were, on average, > 90°). Upright quadrupedal mammals that are of a similar adult size to brown‐throated three‐toed sloths (3.5–5 kg) – including arboreal primates – display limb postures that are relatively more flexed than those measured in brown‐throated three‐toed sloths (Schmitt 1999; Schmidt 2005; Reilly et al. 2007, Schmidt 2008). The more extended elbow and knee postures used by *B. variegatus* during suspensory walking may thusly resemble those of much larger upright mammals (Biewener 1989). Such limb postures may serve to limit the amount of flexor muscle force brown‐throated three‐toed sloths require to support their body weight during suspension (Gray 1968).

Brown‐throated three‐toed sloths displayed significant differences between fore‐ and hindlimb limb kinematics throughout limb support. Overall, elbow angles showed relationships with speed, perhaps consistent with the forelimb serving a primarily propulsive function (McKamy et al. 2023; Young et al. 2023). The significant differences observed between elbow and knee joint angles in brown‐throated three‐toed sloths are not surprising based on their reported morphology, ecology, and behavior. As a genus, *Bradypus* have significantly longer forelimbs in proportion to the hindlimbs, potentially related to adaptations for vertical climbing on larger supports (Jungers 1978; Marshall et al. 2021). Having elongated forelimbs and reduced hindlimb lengths is assumed to minimize the moment the body weight exerts to pitch the animal away from the vertical support while maximizing foot friction during vertical climbing (Jungers 1978). Moving with more extended knee joints may therefore help brown‐throated three‐toed sloths equalize the “functional” length of the fore‐ and hindlimbs. It is known that *B. variegatus* prefer the high canopy/emergent level of the rainforest and is thusly required to navigate a more vertical niche (Urbani and Bosque 2007; Hayssen 2010, 2011).

Brown‐throated three‐toed sloths use non‐parasagittal arm positions when pulling the body forward. The pronounced arm abduction at TD and MS is likely associated with strong laterally directed forces during suspensory walking that are ~10%–20% of body weight (comparable in magnitude to propulsive and braking forces; McKamy et al. 2023). In the sagittal plane, *B. variegatus* use their long, extended forelimbs to increase stride length, specifically showing greater forelimb protraction at TD and increased hindlimb retraction at LO, similar to arboreal primates (Larson et al. 2000, 2001). While limb postures were overall more extended in brown‐throated three‐toed sloths (i.e., elbow and knee angles > 90°), they still depend on flexor musculature activity to maintain joint positions in tension, like all suspensory mammals. Gorvet et al. (2020) found that elbow (*M. biceps brachii* and *M. brachioradialis*) and digital (*M. palmaris longus* and *M. flexor digitorium profundus*) flexors, as well as humeral retractors (*M. deltoideus*, spinal and acromial parts), are active throughout most of the limb support phase during suspensory walking in *B. variegatus*. Nevertheless, the use of relatively more extended limb joints likely mitigates the levels of flexor muscle activity required to maintain joint position and/or limb postures (Biewener 1989), commensurate with low levels of flexor muscle EMG during postural hanging in *Bradypus* (Gorvet et al. 2020).

### 4.1 Divergence of Two‐ Versus Three‐Toed Sloth Limb Kinematics

Despite two‐ and three‐toed sloths sharing patterns of branch support use within a similar arboreal niche (Adam 1999; Hayssen 2009, 2010, 2011), both modern genera arose from separate lineages that split nearly 29 million years ago (see Delsuc et al. 2019 for phylogenetic reconstructions of sloth evolution; Raj Pant et al. 2014). While physiological and morphological traits, as well as arboreal behaviors, represent remarkable examples of evolutionary convergence (e.g., bone cortical compactness, pectoral girdle musculature, and suspensory posture: Nyakatura and Fischer 2011; Raj Pant et al. 2014; Montañez‐Rivera et al. 2018; Alfieri et al. 2021), available data suggest that the two tree sloth radiations show distinct differences in both locomotor kinematics and kinetics, indicative of their evolutionary divergence. Recent studies have shown that, unlike two‐toed sloths, brown‐throated three‐toed sloths display hindlimb‐biased body weight support with significantly greater magnitudes of laterally‐directed forces exerted by both limb pairs (notably the forelimbs) during suspensory locomotion (McKamy et al. 2023). Differences in mediolateral forces are potentially related to differences in how their feet interact with the supports, where brown‐throated three‐toed sloths place the entire volar aspect of their feet in contact with the runway beam (Gorvet et al. 2020; Mendel 1985a) while two‐toed sloths prefer to use mainly their claws to obtain purchase on the support (Granatosky, Karantanis, et al. 2018; Mendel 1981b). Therefore, it is possible that in association with these findings, large degrees of arm abduction and forearm adduction allow for better positioning of the forearm to generate laterally directed forces on the branch support during limb support phase.

**Figure 8:**
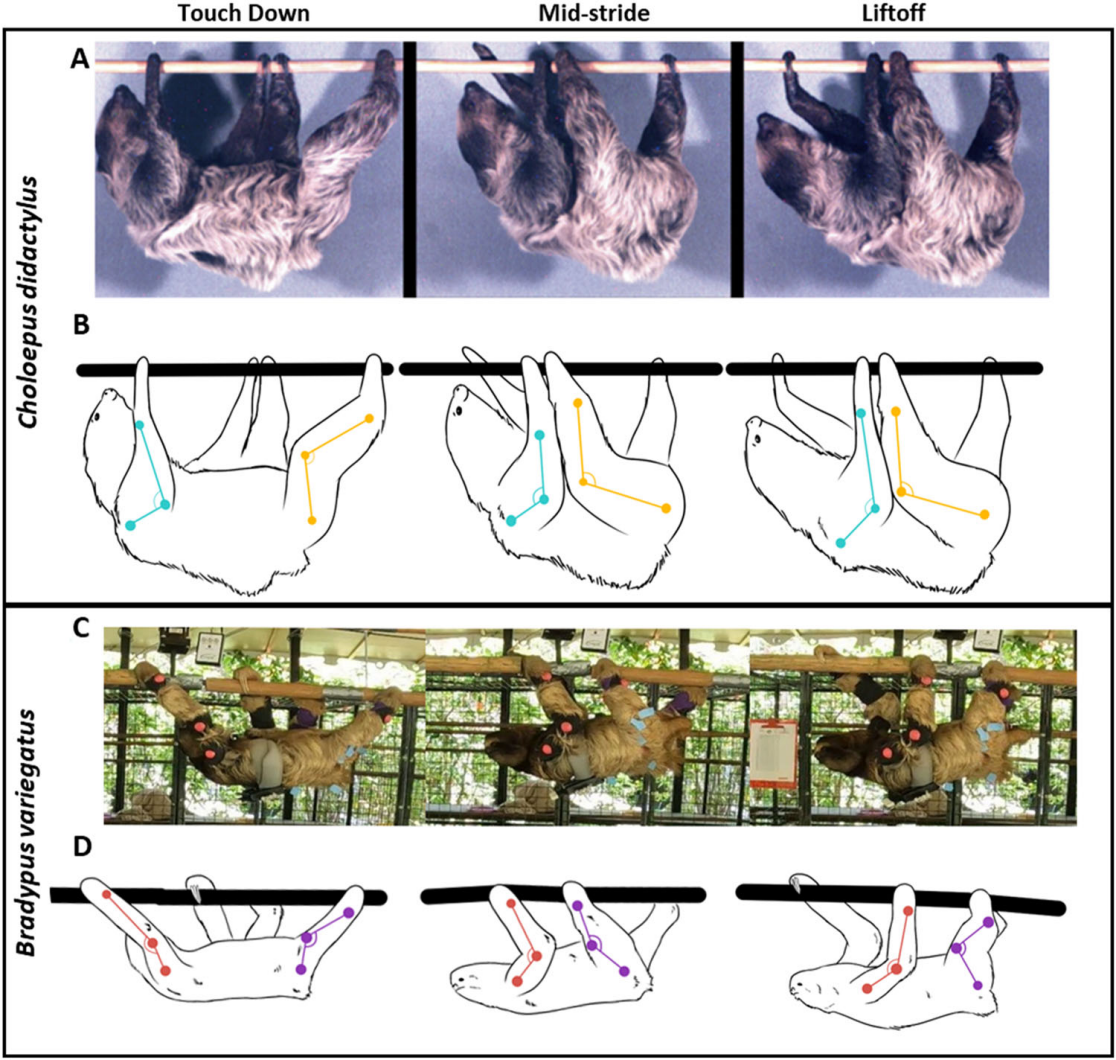
Joint angle comparisons for the fore‐ and hindlimb between *Choloepus didactylus* and *Bradypus variegatus*. Representative joint angles during forelimb tracking at TD, MS, and LO. (A) Images of *Choloepus didactylus* from previous comparative data during X‐ray motion analysis with approximate joint angles in (B). (C) Images of *Bradypus variegatus* during limb tracking with approximate limb angles in (D).

In addition to these established differences in locomotor kinetics, we found significant differences between joint angles between the two extant genera of tree sloths. Brown‐throated three‐toed sloths keep both the elbow and knee significantly more extended throughout the support phase relative to Linnaeus's two‐toed sloth (Figure 8). This difference in joint postures is consistent with known differences in limb proportions between the two species. Three‐toed sloths have elongated forelimbs relative to hindlimbs, whereas two‐toed sloths have limb proportions of roughly equal length (Wislocki 1928; Merit 1985). Accordingly, three‐toed sloths have a much larger intermembral index (IMI: 1.65 ± 0.11) in comparison to two‐toed sloths (IMI: 1.11 ± 0.03) (Marshall et al. 2021). Generally, a larger IMI is indicative of greater forelimb‐biased body weight support and locomotion in primates (i.e., brachiation; Napier 1967). Despite this notion, the index of greater trochanter height and crural index suggest that the morphology of the hindlimbs of three‐toed sloths provides greater mechanical advantage (e.g., for the gluteal muscles: Marshall et al. 2021), potentially reflective of greater body weight support by the hindlimbs during suspensory locomotion and vertical climbing (McKamy et al. 2023). Additionally, whereas two‐toed sloths have a maximum range of motion for elbow extension of 120° (Fujiwara et al. 2011), three‐toed sloths can extend their elbow up to 180° (Nowak 1999). The ranges of elbow joint motions are limited by both muscles/tendons and by joint surface geometry. Both muscle and joint surface morphology reflect a relatively greater range of joint motion in three‐toed sloths than two‐toed sloths (Fujiwara et al. 2011), commensurate with the kinematic data presented in this study. Extended joint postures have been shown to be consistent with both decreased bone stresses and decreased locomotor cost in upright mammals (Biewener 1983; Biewener et al. 2004; Pontzer 2007). Future work might consider the degree to which more extended joint postures provide similar benefits to three‐toed relative to two‐toed sloths.

## 5 Conclusions and Evolutionary Implications

The limb kinematics of brown‐throated three‐toed sloths are overall reflective of their ecological preferences and morphological adaptations. Having proportionally shorter hindlimbs in comparison to forelimbs has led brown‐throated three‐toed sloths to use more extended joint postures, particularly at the knee joint. The alternative strategy to this—increasing elbow flexion, resulting in a functionally shorter forelimb—would increase bone loading and therefore be metabolically expensive, which would not be beneficial to an animal whose success relies on energy conservation. Extended limb postures in sloths are different from above‐branch locomotion in similarly sized quadrupedal arboreal primates, where sloths use greater elbow and knee joint extensions during the limb support phase (Schmidt 2005, 2008). However, during inverted quadrupedal locomotion in some primates, average elbow and knee angles at TD fall in the same range as that of sloths (Dickinson et al. 2022), suggesting greater joint extension may be a more generalized adaptation to inverted quadrupedalism. Additionally, joint angles also significantly differed between two‐ and three‐toed sloths, with brown‐throated three‐toed sloths showing elbow and knee angles that were notably more extended than Linnaeus's two‐toed sloth, potentially reflective of differing limb lengths and preferred arboreal support usage.

The data presented in this study, along with the comparative dynamic analyses by McKamy et al. (2023) and Young et al. (2023), demonstrate that two‐ and three‐toed sloths differ in several functional aspects, including morphology, limb‐loading, and kinematics. Similarly, Dickinson et al. (2022), showed that primates achieve inverted quadrupedal locomotion using a variety of different mechanisms. Dickinson et al. (2022) attributed these findings to the fact that each primate species in their study adopted suspensory walking independently (e.g., analogous to independent evolutionary origins). Given the independent origins of suspensory locomotion in the two extant lineages of tree sloths, it is not surprising that two‐ and three‐toed forms developed distinct strategies to overcome the mechanical demands of inverting the body below branches. However, future comparative analyses are necessary to expand on our understanding of the causes and consequences of sloth kinematic divergence.

## Author Contributions

**Angela M. Mossor:** conceptualization, data curation, formal analysis, investigation, methodology, project administration, resources, software, visualization, writing (original draft, review, editing). **Andrew J. McKamy:** investigation, resources, writing (review, editing). **Melody W. Young:** funding acquisition, investigation, resources, writing (review, editing). **Andrew J. Rochté:** project administration, resources, supervision, writing (review, editing). **Judy A. Avey‐Arroyo:** project administration, resources, supervision, writing (review, editing). **John A. Nyakatura:** data curation, investigation, writing (review, editing). **Michael C. Granatosky:** funding acquisition, investigation, methodology, project administration, resources, writing (review, editing). **Michael T. Butcher:** investigation, methodology, project administration, supervision, resources, writing (review, editing). **Jesse W. Young:** conceptualization, data curation, formal analysis, investigation, methodology, project administration, resources, software, supervision, visualization, writing (original draft, review, editing).

## Conflicts of Interest

The authors declare no conflicts of interest.

## Supporting information

Supporting information.

## Data Availability

The data that support the findings of this study are available from the corresponding author upon reasonable request.
