# Supplementary material for: Three‐Dimensional Limb Kinematics in Brown‐Throated Three‐Toed Sloths (Bradypus variegatus) During Suspensory Quadrupedal Locomotion
Source: J Exp Zool A Ecol Integr Physiol. 2025 Mar 3;343(5):564–77. doi: 10.1002/jez.2911 (PMC12053029; doi:10.1002/jez.2911)
Supplement: Supplementary file 1 — Supporting information. [file JEZ-343-564-s001.pdf]

SUPPLEMENTARY DATA TABLES

Table S1: Statistical comparisons of elbow and knee angles

(A). Elbow Angles

Term	Mean Difference	Statistic	*P**
Overall ANOVA			
Speed	--	$F_{[1,24.705]} = 4.03$	**0.056**
Event	--	$F_{[2,60.325]} = 68.70$	**<0.0001**
Speed-Event Interaction	--	$F_{[2,60.345]} = 1.71$	NS
Pairwise comparisons – Event			
LO - MS	2.67 degrees	$t_{[63.1]} = 1.00$	NS
LO – TD	-25.69 degrees	$t_{[63.1]} = -9.61$	**<0.0001**
MS - TD	-28.37 degrees	$t_{[63.1]} = -10.61$	**<0.0001**

* Significant tests following adjustment are indicated by bold typeface.

NS – not significant

(B). Knee Angles

Term	Mean Difference	Statistic	*P**
Overall ANOVA			
Speed	--	$F_{[1,102.18]} = 3.74$	**0.056**
Event	--	$F_{[2,102.94]} = 25.91$	**<0.0001**
Speed-Event Interaction	--	$F_{[2,100.91]} = 0.037$	NS
Pairwise comparisons – Event			
LO - MS	17.80 degrees	$t_{[103]} = 6.17$	**<0.0001**
LO – TD	-0.018 degrees	$t_{[103]} = -0.006$	NS

MS - TD	-17.819 degrees	$t_{[103]} = -6.27$	**0.0001**

* Significant tests following adjustment are indicated by bold typeface.

NS – not significant

(C). Elbow vs. Knee Elbow Angles: TD

**Term**	**Mean Difference**	**Statistic**	***P****
Overall ANOVA			
Limb	--	$F_{[1]} = 9.68$	**0.0026**
Speed	--	$F_{[1]} = 1.3892$	NS
Limb-Speed Interaction	--	$F_{[1]} = 0.044$	NS
Pairwise comparisons – Limb			
Elbow - Knee	-11.6 degrees	$t_{[74]} = -2.64$	**0.01**

* Significant tests following adjustment are indicated by bold typeface.

NS – not significant

(D). Elbow vs. Knee Elbow Angles: MS

**Term**	**Mean Difference**	**Statistic**	***P****
Overall ANOVA			
Limb	--	$F_{[1]} = 59.20$	**<0.0001**
Speed	--	$F_{[1]} = 0.0299$	NS
Limb-Speed Interaction	--	$F_{[1]} = 1.01$	NS
Pairwise comparisons – Limb			
Elbow - Knee	-25.2 degrees	$t_{[74]} = -7.42$	**<0.0001**

* Significant tests following adjustment are indicated by bold typeface.

NS – not significant

(E). Elbow vs. Knee Elbow Angles: LO

Term	Mean Difference	Statistic	*P**
Overall ANOVA			
Limb	--	$F_{[1]} = 147.52$	**<2^{e-16}**
Speed	--	$F_{[1]} = 0.901$	NS
Limb-Speed Interaction	--	$F_{[1]} = 0.57$	NS
Pairwise comparisons – Limb			
Elbow - Knee	-40.8 degrees	$t_{[72]} = -11.67$	**<0.0001**

* Significant tests following adjustment are indicated by bold typeface.

NS – not significant

Table S2: Statistical comparisons of elbow and knee angles between sloth species

(A). *Bradypus* vs. *Choloepus* Elbow Angles

Term	Mean Difference	Statistic	*P**
Overall ANOVA			
Speed	--	$F_{[1]} = 160.76$	**<0.0001**
Species	--	$F_{[1]} = 139.26$	**<0.0001**
Speed-Species Interaction	--	$F_{[1]} = 0.72$	NS
Pairwise comparisons – Species			
Bradypus - *Choloepus*	52.1 degrees	$t_{[213]} = 11.81$	**<0.0001**

* Significant tests following adjustment are indicated by bold typeface.

NS – not significant

(B). *Bradypus* vs. *Choloepus* Knee Angles

Term	Mean Difference	Statistic	*P**
Overall ANOVA			
Speed	--	$F_{[1]} = 191.65$	**<0.0001**
Species	--	$F_{[1]} = 400.54$	**<0.0001**
Speed-Species Interaction	--	$F_{[1]} = 0.083$	NS
Pairwise comparisons – Species			
Bradypus - *Choloepus*	55.2 degrees	$t_{[196]} = 19.73$	**<0.0001**

* Significant tests following adjustment are indicated by bold typeface.

NS – not significant

(C). *Bradypus* vs. *Choloepus* Elbow Angles: TD

**Term**	**Mean Difference**	**Statistic**	***P****
Overall ANOVA			
Speed	--	$F_{[1]} = 86.84$	**<0.0001**
Species	--	$F_{[1]} = 86.95$	**<0.0001**
Speed-Species Interaction	--	$F_{[1]} = 0.0024$	NS
Pairwise comparisons – Species			
Bradypus - *Choloepus*	72.7 degrees	$t_{[73]} = 9.27$	**<0.0001**

* Significant tests following adjustment are indicated by bold typeface.

NS – not significant

(D). *Bradypus* vs. *Choloepus* Elbow Angles: MS

**Term**	**Mean Difference**	**Statistic**	***P****
Overall ANOVA			
Speed	--	$F_{[1]} = 96.69$	**<0.0001**
Species	--	$F_{[1]} = 84.69$	**<0.0001**
Speed-Species Interaction	--	$F_{[1]} = 1.41$	NS
Pairwise comparisons – Species			
Bradypus - *Choloepus*	50.5 degrees	$t_{[66]} = 9.27$	**<0.0001**

* Significant tests following adjustment are indicated by bold typeface.

NS – not significant

(E). *Bradypus* vs. *Choloepus* Elbow Angles: LO

**Term**	**Mean Difference**	**Statistic**	***P****
Overall ANOVA			
Speed	--	$F_{[1]} = 57.90$	**<0.0001**
Species	--	$F_{[1]} = 31.60$	**<0.0001**
Speed-Species Interaction	--	$F_{[1]} = 0.13$	NS
Pairwise comparisons – Species			
Bradypus - *Choloepus*	31.9 degrees	$t_{[66]} = 5.57$	**<0.0001**

* Significant tests following adjustment are indicated by bold typeface.

NS – not significant

(F). *Bradypus* vs. *Choloepus* Knee Angles: TD

**Term**	**Mean Difference**	**Statistic**	***P****
Overall ANOVA			
Speed	--	$F_{[1]} = 51.18$	**<0.0001**
Species	--	$F_{[1]} = 167.08$	**<0.0001**
Speed-Species Interaction	--	$F_{[1]} = 0.0013$	NS
Pairwise comparisons – Species			
Bradypus - *Choloepus*	47.4 degrees	$t_{[63]} = 12.86$	**<0.0001**

* Significant tests following adjustment are indicated by bold typeface.

NS – not significant

(G). *Bradypus* vs. *Choloepus* Knee Angles: MS

**Term**	**Mean Difference**	**Statistic**	***P****
Overall ANOVA			
Speed	--	$F_{[1]} = 93.67$	**<0.0001**
Species	--	$F_{[1]} = 155.91$	**<0.0001**
Speed-Species Interaction	--	$F_{[1]} = 0.0012$	NS
Pairwise comparisons – Species			
Bradypus - *Choloepus*	47.4 degrees	$t_{[63]} = 12.20$	**<0.0001**

* Significant tests following adjustment are indicated by bold typeface.

NS – not significant

(H). *Bradypus* vs. *Choloepus* Knee Angles: LO

**Term**	**Mean Difference**	**Statistic**	***P****
Overall ANOVA			
Speed	--	$F_{[1]} = 173.30$	**<0.0001**
Species	--	$F_{[1]} = 350.00$	**<0.0001**
Speed-Species Interaction	--	$F_{[1]} = 0.048$	NS
Pairwise comparisons – Species			
Bradypus - *Choloepus*	71.9 degrees	$t_{[62]} = 18.41$	**<0.0001**

* Significant tests following adjustment are indicated by bold typeface.

NS – not significant

Table S3: Statistical comparisons of arm and thigh abduction

(A). Arm Abduction

Term	Mean Difference	Statistic	*P**
Overall ANOVA			
Speed	--	$F_{[1,33.95]} = 0.092$	NS
Event	--	$F_{[2,44.51]} = 6.81$	**0.0026**
Speed-Event Interaction	--	$F_{[2,44.92]} = 0.44$	NS
Pairwise comparisons – Event			
LO - MS	-1.61 degrees	$t_{[43.5]} = -3.58$	**0.0024**
LO – TD	-0.76 degrees	$t_{[44.3]} = -1.69$	NS
MS - TD	0.85 degrees	$t_{[43.5]} = 2.21$	NS

* Significant tests following adjustment are indicated by bold typeface.

NS – not significant

(B). Thigh Abduction

Term	Mean Difference	Statistic	*P**
Overall ANOVA			
Speed	--	$F_{[1,53]} = 0.026$	NS
Event	--	$F_{[2,53]} = 7.12$	**0.0018**
Speed-Event Interaction	--	$F_{[2,49.40]} = 1.10$	NS
Pairwise comparisons – Event			
LO - MS	1.20 degrees	$t_{[52.4]} = 2.43$	NS
LO – TD	2.61 degrees	$t_{[51.8]} = 3.43$	**0.0033**
MS - TD	1.41 degrees	$t_{[52.6]} = 1.87$	NS

* Significant tests following adjustment are indicated by bold typeface.

NS – not significant

(C). Arm vs. Thigh Abduction: TD

Term	Mean Difference	Statistic	*P**
Overall ANOVA			
Limb	--	$F_{[2]} = 45.86$	**<0.0001**
Speed	--	$F_{[1]} = 1.08$	NS
Limb-Speed Interaction	--	$F_{[2]} = 1.98$	NS
Pairwise comparisons – Limb			
Arm - Thigh	18.8 degrees	$t_{[118]} = 8.18$	**<0.0001**

* Significant tests following adjustment are indicated by bold typeface.

NS – not significant

(D). Arm vs. Thigh Abduction: MS

Term	Mean Difference	Statistic	*P**
Overall ANOVA			
Limb	--	$F_{[2]} = 13.34$	**<0.0001**
Speed	--	$F_{[1]} = 2.78$	NS
Limb-Speed Interaction	--	$F_{[2]} = 1.45$	NS
Pairwise comparisons – Limb			
Arm - Thigh	11.4 degrees	$t_{[118]} = 4.55$	**<0.0001**

* Significant tests following adjustment are indicated by bold typeface.

NS – not significant

(E). Arm vs. Thigh Abduction: LO

Term	Mean Difference	Statistic	*P**
Overall ANOVA			
Limb	--	$F_{[2]} = 1.30$	NS
Speed	--	$F_{[1]} = 1.44$	NS
Limb-Speed Interaction	--	$F_{[2]} = 0.41$	NS
Pairwise comparisons – Limb			
Arm - Thigh	-7.08 degrees	$t_{[93]} = -1.33$	NS

* Significant tests following adjustment are indicated by bold typeface.

NS – not significant

Table S4: Statistical comparisons of forearm and leg abduction

(A). Forearm Abduction

Term	Mean Difference	Statistic	*P**
Overall ANOVA			
Speed	--	$F_{[1,34.34]} = 0.77$	NS
Event	--	$F_{[2,60.77]} = 4.80$	**0.012**
Speed-Event Interaction	--	$F_{[2,60.59]} = 0.35$	NS
Pairwise comparisons – Event			
LO - MS	-2.72 degrees	$t_{[60.9]} = -1.84$	NS
LO – TD	-4.50 degrees	$t_{[60.9]} = -3.05$	**0.0094**
MS - TD	-1.78 degrees	$t_{[59.1]} = -1.35$	NS

* Significant tests following adjustment are indicated by bold typeface.

NS – not significant

(B). Leg Abduction

Term	Mean Difference	Statistic	*P**
Overall ANOVA			
Speed	--	$F_{[1,13.12]} = 1.14$	NS
Event	--	$F_{[2,46.17]} = 9.82$	**0.0003**
Speed-Event Interaction	--	$F_{[2,43.90]} = 0.42$	NS
Pairwise comparisons – Event			
LO - MS	7.01 degrees	$t_{[47.7]} = 3.30$	**0.0051**
LO – TD	9.09 degrees	$t_{[47.7]} = 4.28$	**0.0003**
MS - TD	2.08 degrees	$t_{[47]} = 1.10$	NS

* Significant tests following adjustment are indicated by bold typeface.

NS – not significant

(C). Forearm vs. Leg Abduction: TD

Term	Mean Difference	Statistic	*P**
Overall ANOVA			
Limb	--	$F_{[2]} = 3.50$	**0.033**
Speed	--	$F_{[1]} = 0.44$	NS
Limb-Speed Interaction	--	$F_{[2]} = 0.06$	NS
Pairwise comparisons – Limb			
Forearm - Leg	5.50 degrees	$t_{[118]} = 2.32$	NS

* Significant tests following adjustment are indicated by bold typeface.

NS – not significant

(D). Forearm vs. Leg Abduction: MS

Term	Mean Difference	Statistic	*P**
Overall ANOVA			
Limb	--	$F_{[2]} = 0.0043$	NS
Speed	--	$F_{[1]} = 3.48$	NS
Limb-Speed Interaction	--	$F_{[2]} = 0.88$	NS
Pairwise comparisons – Limb			
Forearm - Leg	0.069 degrees	$t_{[118]} = 0.032$	NS

* Significant tests following adjustment are indicated by bold typeface.

NS – not significant

(E). Forearm vs. Leg Abduction: LO

Term	Mean Difference	Statistic	*P**
Overall ANOVA			
Limb	--	$F_{[2]} = 13.14$	**<0.0001**
Speed	--	$F_{[1]} = 0.0001$	NS
Limb-Speed Interaction	--	$F_{[2]} = 0.33$	NS
Pairwise comparisons – Limb			
Forearm - Leg	-10.06 degrees	$t_{[93]} = -4.42$	**0.0001**

* Significant tests following adjustment are indicated by bold typeface.

NS – not significant

Table S5: Statistical comparisons of fore- and hindlimb protraction

(A). Forelimb Protraction

Term	Mean Difference	Statistic	*P**
Overall ANOVA			
Speed	--	$F_{[1,28.02]} = 2.87$	NS
Event	--	$F_{[2,61.29]} = 151.63$	**<0.0001**
Speed-Event Interaction	--	--	NS
Pairwise comparisons – Event			
LO - MS	-21.8 degrees	$t_{[63.4]} = -7.45$	**<0.0001**
LO – TD	-50.7 degrees	$t_{[63.4]} = -17.36$	**<0.0001**
MS - TD	-28.9 degrees	$t_{[63.4]} = -9.90$	**<0.0001**

* Significant tests following adjustment are indicated by bold typeface.

NS – not significant

(B). Hindlimb Protraction

Term	Mean Difference	Statistic	*P**
Overall ANOVA			
Speed	--	$F_{[1,68.28]} = 1.72$	NS
Event	--	$F_{[2,68.01]} = 399.78$	**<0.0001**
Speed-Event Interaction	--	--	NS
Pairwise comparisons – Event			
LO - MS	-37.4 degrees	$t_{[47.4]} = -17.79$	**<0.0001**
LO – TD	-58.7 degrees	$t_{[47.4]} = -27.94$	**<0.0001**
MS - TD	-21.3 degrees	$t_{[46.2]} = -10.41$	**<0.0001**

* Significant tests following adjustment are indicated by bold typeface.

NS – not significant

(C). Forelimb vs. Hindlimb Protraction: TD

**Term**	**Mean Difference**	**Statistic**	***P****
Overall ANOVA			
Limb	--	$F_{[1]} = 5.48$	**0.022**
Speed	--	$F_{[1]} = 19.27$	**<0.0001**
Limb-Speed Interaction	--	$F_{[1]} = 0.28$	NS
Pairwise comparisons – Limb			
Forelimb - Hindlimb	8.32 degrees	$t_{[74]} = 3.50$	**0.0008**

* Significant tests following adjustment are indicated by bold typeface.

NS – not significant

(D). Forelimb vs. Hindlimb Protraction: MS

**Term**	**Mean Difference**	**Statistic**	***P****
Overall ANOVA			
Limb	--	$F_{[1]} = 078$	NS
Speed	--	$F_{[1]} = 1.34$	NS
Limb-Speed Interaction	--	$F_{[1]} = 0.001$	NS
Pairwise comparisons – Limb			
Forelimb - Hindlimb	0.039 degrees	$t_{[74]} = 0.51$	NS

* Significant tests following adjustment are indicated by bold typeface.

NS – not significant

(E). Forelimb vs. Hindlimb Protraction: LO

Term	Mean Difference	Statistic	***P****
Overall ANOVA			
Limb	--	$F_{[1]} = 17.16$	**<0.0001**
Speed	--	$F_{[1]} = 0.402$	NS
Limb-Speed Interaction	--	$F_{[1]} = 0.055$	NS
Pairwise comparisons – Limb			
Forelimb - Hindlimb	-13.6 degrees	$t_{[72]} = 4.15$	**0.0001**

* Significant tests following adjustment are indicated by bold typeface.

NS – not significant

Table S6. Forelimb vs. Hindlimb Excursion

Term	Mean Difference	Statistic	*P**
Overall ANOVA			
Speed	--	$F_{[1,71.670]} = 7.84$	**0.0066**
Limb	--	$F_{[1,68.43]} = 2.46$	NS
Speed-Limb Interaction	--	--	NS

*To control for multiple simultaneous comparisons, *p*-values for post hoc comparisons were adjusted using the false discovery rate method (Benjamini and Hochberg, 1995). Significant tests following adjustment are indicated by bold typeface.

NS – not significant
